# Highly variable rate of orthodontic tooth movement measured by a novel 3D method correlates with gingival inflammation

**DOI:** 10.1007/s00784-020-03502-2

**Published:** 2020-08-18

**Authors:** Marc A. de Gouyon Matignon de Pontouraude, Johannes W. Von den Hoff, Frank Baan, Robin Bruggink, Marjon Bloemen, Ewald M. Bronkhorst, Edwin M. Ongkosuwito

**Affiliations:** 1 Department of Dentistry, section of Orthodontics and Craniofacial Biology, Radboud University Nijmegen Medical Centre, Nijmegen, The Netherlands; 2 3D Lab, Radboud University Nijmegen Medical Centre, Nijmegen, The Netherlands

**Keywords:** Intra-oral scan, Gingival crevicular fluid, Inflammation, Matrix metalloproteinases, Orthodontics, Three-dimensional imaging

## Abstract

**Objectives:**

Individual orthodontic treatment duration is hard to predict. Individual biological factors are amongst factors influencing individual rate of orthodontically induced tooth movement (OTM). The study aim is to determine the rate of OTM by a novel 3D method and investigate parameters that may predict the rate of tooth movement.

**Materials and methods:**

In this prospective cohort study, rate of OTM was determined from 90 three-dimensional intra-oral scans in 15 patients (aged 12–15) undergoing orthodontic treatment. For each patient, intra-oral scans were taken every week for up to 6 weeks (T0–T5). The teeth were segmented from the scans and the scans were superimposed on the palatal rugae. The rate of OTM was calculated for each tooth. Other parameters were gingival inflammation, contact-point displacement and the biological markers, matrix metalloproteinases (MMP), MMP-9 and MMP-2 in gingival crevicular fluid (GCF).

**Results:**

Our study showed a high variation in the rate of OTM, varying from 0.15 to 1.24 mm/week. Teeth in the anterior segment tended to move more compared with the posterior segment. The contact point displacement and gingival inflammation varied greatly amongst the patients. The MMPs measured did not correlate with tooth movement. However, the gingival inflammation index showed a significant correlation with OTM. Future studies should include other biological markers related to bone-remodeling.

**Conclusion:**

This novel and efficient 3D method is suitable for measuring OTM and showed large individual variation in rate of OTM.

**Clinical relevance:**

Patients show different rates of OTM. The rate of OTM in an individual patient can provide guidance in timing of follow-up appointments.

## Introduction

Improvement of dental function and aesthetics with orthodontic treatment is becoming increasingly popular. In 2011, 60% of all Dutch young adults between 17 and 23 years old had orthodontic treatment during their adolescence [1]. For many people undergoing orthodontic treatment, the treatment duration is a major drawback [2]. Treatment should be of high quality, cost-effective and as short as possible, to limit the risk of iatrogenic side effects such as white spot lesions [3]. The individual treatment duration is still hard to predict. Treatment duration is influenced by several factors of which individual rate of orthodontically induced tooth movement (OTM) probably plays an important role. In beagle dogs, it is shown that individual rate of OTM is highly variable [4, 5]. Clinical studies show OTM rates between 0.55 and 2.44 mm per month when using full fixed appliances and NiTi wires for initial alignment [6].

In general, orthodontic tooth movement is divided into four phases; the initial phase, the hyalinization phase, the acceleration phase and the linear phase [7, 8]. Extensive remodeling of alveolar bone and the periodontal ligament is a prerequisite for OTM [7]. In the gingival crevicular fluid (GCF), biological components of the remodeling process can be measured [9, 10]. Cytokines and other regulatory proteins are present in the GCF and provide information on periodontal health and the process of tissue remodeling. Pro-inflammatory cytokines such as IL-1ß, TNF-α and IL-6 have been identified in the GCF of orthodontic patients as well as PGE_2_ [11, 12]. Some studies show a temporary up-regulation of IL-1ß after 24 h of force application [13]. Others demonstrated continuous elevated IL-1ß levels starting from 24 h [14]. PGE_2_ was found to be elevated after 24 h in most studies [14].

A specific group of proteases that play a key role in tissue remodeling during OTM is the matrix metalloproteinases (MMPs) [15]. MMPs degrade extracellular matrix (ECM) components during physiological matrix turnover [9]. The collagen triple helix is degraded to gelatin by the collagenases, MMP-1, -8 and -13. Subsequently, the gelatinases MMP-2 and MMP-9 degrade the gelatin [9]. The other MMPs more preferentially degrade other matrix proteins. Techniques such as zymography and enzyme-linked immunosorbent assay (ELISA) can be used to analyze MMPs [16]. Zymography is a highly sensitive technique to identify and quantify MMPs. Experimental animal and human studies have shown increased levels of MMP-1, -2, -8, -9 and -13 in GCF during OTM [17–19]. However, these studies did not compare the MMP levels with the rate of OTM.

Conventionally, OTM is measured through superimposition of plaster cast models or sequential cephalograms. With plaster cast models, differences are often limited to pre- and post-treatment models only, as multiple impression taking can be a burden to the patient [20]. Cephalograms lack a third dimension. Other shortcomings of this method are the radiation exposure, tracing difficulties due to overlapping structures and magnification errors [21]. Using 2D measurements, it is impossible to determine whether the teeth moved bodily or display angulations, rotations and inclinations [21]. By using a 3D intra-oral scanner, weekly scans are feasible and can overcome these limitations and reduce the radiation dose.

We hypothesize that by using this novel method, the individual rate of OTM can be determined and that the rate of OTM is correlated to the level of MMPs in GCF or to other individual patient characteristics.

## Materials and methods

### Study design

This prospective cohort study compared the effects of orthodontic tooth alignment with several patient-related and biological parameters. This study was approved by the medical ethics committee (2015-2213, CMO, Arnhem-Nijmegen, The Netherlands). Written informed consent was received from all parents, guardians and children.

### Setting

Participants fulfilling the inclusion criteria were recruited from the orthodontic university clinic of the Radboud University Medical Centre in Nijmegen starting from April 2016. Follow-up occurred till June 2017 and covered appliance placement up to completion of alignment. Fourteen patients are required to have a 90% chance of detecting, as significant at the 5% level, an increase in the primary outcome measure from 0.6 in the control group to 3.05 in the experimental group [22, 23].

### Participants

All patients were in good general health, without craniofacial malformations. Inclusion criteria included the following: (1) no use of medication, (2) no radiographic evidence of periodontal bone loss, (3) no syndromes, (4) no agenetic missing teeth or other dental developmental malformations and (5) no dental extractions. Fixed appliances and bonding method were standardized (GC Orthodontics, Experience Metal, 0.022-inch self-ligating brackets, Roth prescription, Breckerfeld, Germany). After bracket bonding, a 0.014-inch beta-titanium archwire (GC Orthodontics, Breckerfeld, Germany) was ligated. This wire delivers a continuous force of approximately 150 cN [24]. The archwire was cut distal to the first molar teeth and not cinched. No bite planes, auxiliary arches, intermaxillary elastics, headgears or temporary anchorage devices were used during the study. All appliances were placed by postgraduate orthodontic trainees under direct supervision of a consultant orthodontist. The Silness-Löe (S&L) index was used as an indicator for inflammation and scored every week [25]. Additionally for each patient, the severity of contact-point displacement was scored by Little’s irregularity index (LII) [26]. LII is used in orthodontics to address the severity of the malocclusion and predict duration of orthodontic treatment.

### Collection and superimposition of 3D scans

All participants were scanned intra-orally using a chair-side intraoral Trios3 scanner and software ((version 1.18.1.3) 2014 3Shape, Copenhagen, Denmark). At the start of the treatment (T0), intraoral scans of both upper and lower arch were taken every week up to 4 weeks after placement, as part of our clinical protocol. Lower jaw scans were not used in this study. The last scan used (T5) was taken at the first check-up appointment (6 weeks after placement), when wires were changed. Patients were sitting in the dental chair inclined at 45°. Their teeth were gently dried with air, and the cheeks were retracted for scanning and moisture control. The teeth were continuously scanned from one side of the posterior teeth to the opposite side along the dental arch: initially the occlusal surface, then the buccal and lingual surfaces and finally the palatal area. The scanning procedure took approximately 10 min per patient. Previous studies have already confirmed the accuracy and validity of digital models created with this intraoral scanner [27–29].

**Fig. 1:**
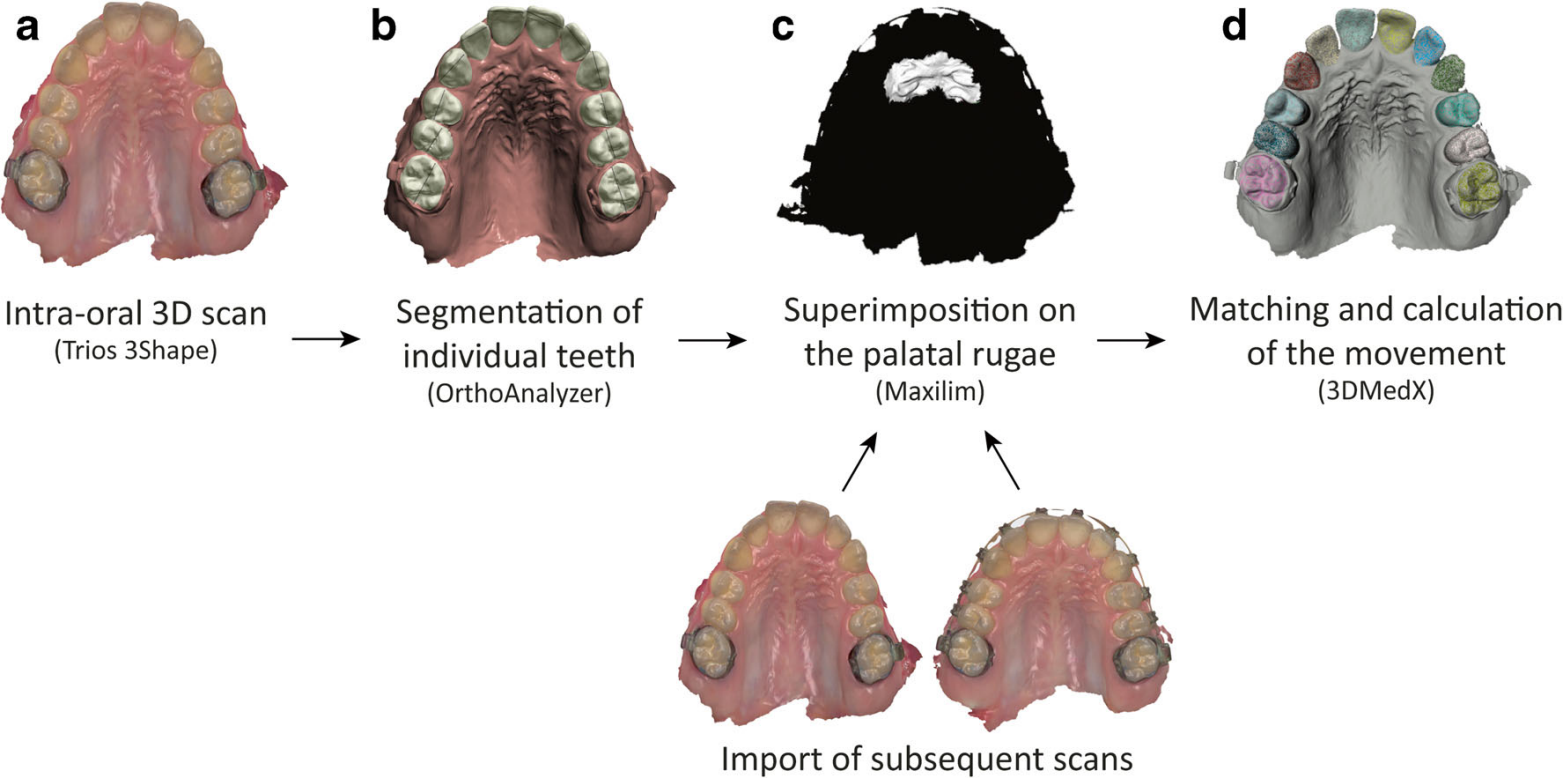
Steps to generate, segment, superimpose and analyze intra-oral scans. **a** The dentition of the patient is scanned with an intra-oral scanner. **b** The teeth are segmented as separate objects. **c** Superimposition of two subsequent scans on the palatal rugae. **d** Matching of the teeth on the subsequent scan to calculate movement

The analysis consisted of four steps (Fig. 1). The scan taken of the upper jaw at T0 was segmented into separate teeth using OrthoAnalyzer software ((version 1.7.1.1) 3Shape, Copenhagen, Denmark). All scans were imported as .STL files into Maxilim software ((version 2.3.0.3) Medicim N.V., Mechelen, Belgium). Surface-based superimpositions of the scans were done on the palatal rugae, since these are considered most stable during OTM [21, 30–32]. Four reference points were placed on the rugae of each palate. After this initial check, a distance map with color coding was created using an Iterative Closest Point matching to assess the stability of the area for superimposition. Between each time point, distance kits were created to assess stability of the palatal rugae. All scans and individually segmented teeth were then imported into custom made software (3DMedX) that was developed with C++ in Microsoft Visual Studio 2015 ((version 14.0) Microsoft Corporation, Redmond, WA, USA) [33]. This easy-to-use software moved the segmented teeth from the imported T0 scan to the position of the teeth within the scans taken at later time points using surface-based registration as described by Baan et al. [34]. Clinically relevant information, such as anterior/posterior, left/right and up/down movements were given as output by the software. All movement was measured in millimeters. The maxillary teeth were divided in three groups, the frontal or 2nd segment consisting of the anterior six teeth. The lateral segments, or 1st and 3rd segment, consist of the left and right posterior teeth. Since left and right posterior teeth have the same root anatomy and all patients had a symmetrical dental arch, left and right were combined in the analysis.

### GCF sampling

GCF samples were collected from all teeth in the 2nd segment on the buccal and lingual side, and then pooled. The teeth were gently dried with air and a cotton roll. Then, a 1-μl microsyringe (Hamilton, Reno, Nevada, USA) was carefully inserted into the sulci. Starting at T0, before placement of the appliance, the GCF samples were taken every week until 4 weeks after placement and at the first check-up appointment (6 weeks after placement). Sampling was performed just before a visit with all orthodontic appliances in situ. The GCF samples were put on ice immediately and stored at − 20 °C until analysis by gelatin zymography.

### Gelatin zymography

Gelatinases (MMP-2 and -9) in the samples were analyzed by gelatin zymography. The polyacrylamide gel (7.5%) contained 1 mg/ml gelatin, as described by Bildt et al. (2009). Precision Plus Protein Standards (Bio-Rad Laboratories, Hercules, California, USA) ranging from 10 to 250 kDa were included to determine the molecular weight of the MMPs. A (1:1) mixture of the sample and sample buffer was then electrophoresed for 15 min at 60 mA and then at 120 mA until the 37 kDa marker band was no longer visible on the gel. Recombinant human pro-MMP-9 (Oncogene, CN Biosciences, San Diego, California, USA) was used as a reference sample. After electrophoresis, the gels were washed in 2.5% Triton X-100 (Sigma-Aldrich, St. Louis, Missouri, USA) buffer to remove the SDS and the marker bands were cut out to ensure visibility after staining. The gels were then incubated in a second washing buffer containing 50 mM Tris–HCl (pH 7.8), 5 mM CaCl_2_, and 0.1% Triton X-100 at 37 °C for 18 h. They were finally stained for 45 min with 2.5 g/l Coomassie Brillantblue R250 (Imperial Chemical Industries Plc, London, UK) in 10% acetic acid and 40% methanol in water and thereafter destained with 10% acetic acid and 40% methanol in water. The MMPs appear as bright bands within the stained gel. The gels were photographed with a Gel Doc™ EZ Imager and Image Lab™ software ((version 4.1) Bio-Rad Laboratories, Hercules, California, USA). The bands were analyzed with ImageJ software ((version 1.15n) Wayne Rasband, National Institutes of Health, USA). The pro-MMP-9 reference sample enabled comparison of the corresponding bands on different gels. The amount of enzyme in the bands was represented as average density to the reference sample. First, within each gel, the amount of enzyme in the reference sample was arbitrarily set to 1, and all other bands were calculated relative to it. The relative amounts of MMPs were corrected for the total protein concentration in the sample. The protein concentration was determined with a BCA assay.

### BCA assay

The bicinchoninic acid (BCA) assay is a colorimetric assay. A micro BCA protein assay kit (Thermo Scientific Pierce, Rockford, Illinois, USA) was used to determine the protein concentration in the GCF samples. A working reagent and a standard series of bovine serum albumin (BSA) were prepared according to the manufacturers’ protocol (Thermo Scientific Pierce, Rockford, Illinois, USA). For preparation of the calibration curve, a stock solution of BSA (400 μg/ml) was used and then diluted according to manufacturers’ protocol and distributed in a 96-well plate. The GCF samples were diluted with PBS in a 1:100 ratio. Following this, 100-μl sample and 100-μl working reagent per well were added to the plate. The plate was then incubated for 2 h at 37 °C and absorption was measured at 570 nm. The protein concentration was calculated from the protein standard curve.

### Statistical analysis

In order to validate and evaluate the accuracy of tooth movement, one observer reanalyzed 20 models again after a 2-week interval to assess the intra-observer variability. A second observer matched model sets of four patients independently to determine the inter-observer variability (ICC was considered as < 0.40 poor, 0.40–0.59 fair, 0.60–0.74 good and 0.75–1.00 excellent). The teeth in the maxilla were divided into three segments; last molar to first pre-molar, canine to canine and first pre-molar to last molar. The mean and absolute mean differences of the tooth displacement were calculated per segment.

To analyze the effect of biological and patient-related variables on tooth displacement, multiple regression analyses models were used. As data from the consecutive steps in time are clustered within patients, these models were chosen to be random effect models. The analyses were done using the lme4 library for the package R [35].

## Results

### Patient characteristics

A total of 15 orthodontic patients (8 males, 7 females), aged 12.5–15.2 (mean age 13.7 ± 0.8) were included. Five patients were excluded since they missed several follow-up appointments. From each patient, six (T0–T5) intra oral scans were made providing in total 90 intra oral scans. Tooth movement was defined as displacement in three dimensions (*x*, *y* and *z*) in mm. Table 1 shows the characteristics of the included patients. The LII varied from 1.4 to 9.1 mm within the patient group. Patients maintained good oral health during the experiment, which is indicated by the mean S&L index of around 1.03.

**Table 1:** Patient characteristics, *n* = 15

	Mean ± SD	Minimum	Maximum
Age (year)	13.7 ± 0.8	12.5	15.2
LII (mm)	3.93 ± 2.2	1.4	9.1
S&L index	1.03 ± 0.2	0.58	1.58
Matrix metalloproteinases			
Pro-MMP-9	0.80 ± 0.2	0.06	1.23
MMP-2-complex	0.24 ± 0.1	0.01	0.46
Multimer-MMP-9	0.18 ± 0.1	0.08	0.31
Orthodontic tooth movement (mm/week)			
Frontal segment	0.41 ± 0.2	0.15	1.08
Lateral segments	0.40 ± 0.2	0.17	1.24

The movement of the frontal segment is based on the 2nd segment, in mm per tooth per week. The lateral segment is based on the first and third segment combined. The MMP values are relative to the reference value. In this study, a reference value of 1.0 Pro-MMP-9 is equal to 4 μg/5μl

The mean level of MMPs measured varied greatly between patients as shown by the large standard deviation (SD) amongst patients. The amount of tooth movement differed greatly among patients, ranging from 0.15 up to 1.24 mm/week. As shown by the LII, the contact-point displacement and malocclusion were highly variable as well (Table 1).

### Tooth movement

**Fig. 2:**
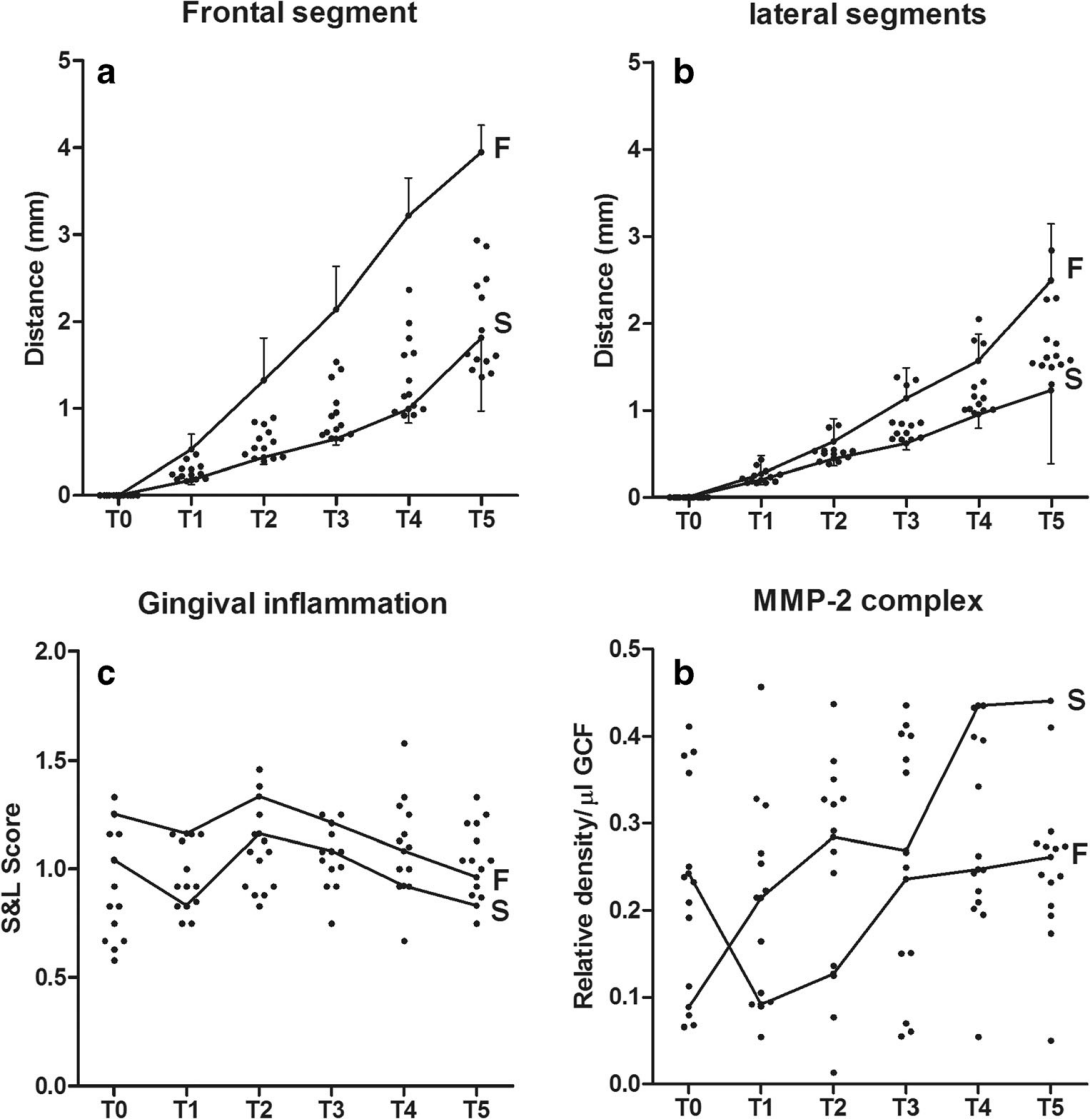
Tooth movement and associated parameters. **a** Cumulative average OTM (mm ± SD) displayed per patient for the frontal segment consisting of the six anterior upper teeth. **b** Cumulative average OTM (mm ± SD) displayed per patient for the lateral segments consisting of both left and right posterior teeth. **c** Gingival inflammation scored using the Silness&Loë index per patient per time point. **d** MMP-2 complex per patient per time point in pooled samples from all analyzed teeth. T indicates the time points. For comparison, the data from the fastest (F) and slowest (S) patients are indicated

Figure 2 shows the tooth movement of the frontal segment and lateral segments of 15 patients during the initial 6 weeks of orthodontic treatment (Fig. 2a, b). The pattern and amount of tooth movement were not constant and showed a large variation amongst patients. Teeth in the frontal segment showed more movement compared with the lateral segment. However, patients that started with slow moving teeth were still “slow movers” at T5 and patients with fast moving teeth at T0 were still fast movers at T5. Table 2 shows the fixed effects of the linear mixed effect model for OTM of the frontal segment and its relationship to the individual patient characteristics. The rate of OTM did not correlate to Little’s irregularity index or the level of MMPs (Pro-MMP-9, MMP-2-complex or Multimer-MMP-9 in GCF). MMP-2-complex showed a positive relation, but this was not significant. The rate of OTM did correlate statistically significantly to the S&L index (0.244 [0.005...0.476]) indicating that inflammation stimulates tooth movement (Table 2). Other than S&L, no significant relations were found. The random effects of this model indicate great individual variation between patients (data not shown). The interclass correlation coefficient in our dataset was 0.385, confirming this large individual variation shown in Fig. 2.

### Gelatin zymography

**Fig. 3:**
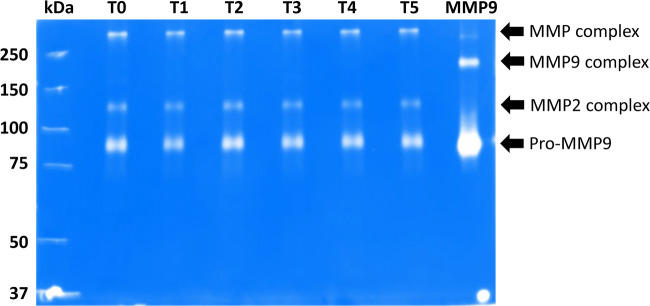
Representative example of zymography. Pooled samples from one of the patients were run on a acrylamide gel containing gelatin as a substrate. At the left side, the molecular weights (kDa) of the standard proteins are indicated. The next six lanes show the samples taken at the different recalls. The most right lane contains the recombinant MMP9 standard with a band for pro-MMP9 and MMP9 complex (arrows). The other arrows indicate two other MMP forms

The gelatin zymograms show that GCF contained gelatinolytic activity in bands around 265, 200, 132 and 92 kDa (Fig. 3). The reference sample indicated that the bands at 200 and 92 kDa were MMP-9 dimer and pro-MMP-9, respectively. The band at 132 kDa was identified as complexed MMP-2, and the band at 265 kDa was most likely a multimer of MMP-9 as described by Bildt et al. (2009).

### Inter- and intra-observer agreement

Since this is a novel method to measure OTM, reliability and reproducibility are essential. Twenty models were reanalyzed by one observer and a second observer matched model sets of four patients independently to determine the intra- and inter-observer variability. The inter-observer reliability was good (0.70 to 0.79) in the *X*, *Y* and *Z* translation of the segments. The intra-observer reliability was excellent (0.92 to 0.99) in the *X*, *Y* and *Z* translation of the segments (see Table 3).

**Table 2:** The rate of OTM relating to individual patient characteristics (linear mixed effects model-fixed effects)

	Effect	*p* value	95% Confidence interval
Intercept	0.049	0.755	[− 0.300...0.413]
LII (mm)	0.023	0.172	[− 0.010...0.056]
S&L index	0.244	0.046*	[0.005...0.476]
Matrix metalloproteinases			
Pro-MMP-9	− 0.018	0.941	[− 0.362...0.336]
MMP-2-complex	0.196	0.713	[− 0.579...0.846]
Multimer-MMP-9	− 0.083	0.897	[− 0.076...0.943]

Significance was set at *α* = 0.05; significant values are indicated with *

## Discussion

To our knowledge, this is the first study that attempts to relate 3D rate of tooth movement to biological factors to determine individual variation with an easy-to-use novel software method. This study shows that high individual differences in OTM exist and this may give opportunities in the near future to personalize orthodontic treatment duration having direct clinical implications. We hypothesized that the rate of OTM could be determined with our 3D method and would correlate to the level of MMPs in GCF or to other individual patient characteristics. As observed, the S&L index does have a predictive value for the rate of OTM. The other parameters measured were not significantly correlated to the rate of OTM.

**Table 3:** Intra-class coefficient for *X*, *Y*, *Z* translations for intra- and inter-observer reliability

	Intra-observer	Inter-observer
X-translation 14-17	0.95	0.76
Y-translation 14-17	0.99	0.71
Z-translation 14-27	0.92	0.78
X-translation 13-23	0.96	0.75
Y-translation 13-23	0.94	0.71
Z-translation 13-23	0.99	0.70
X-translation 24-27	0.97	0.78
Y-translation 24-27	0.99	0.79
Y-translation 24-27	0.96	0.78

The data acquired by this study on the rate of OTM was obtained by 3D superimposition of intra-oral scans. This is an accurate method, using the palatal rugae as reference [32]. Weekly measurements ensure that also small movements are captured. Measuring the individual teeth movement was a process which has a low variability (ICC > 0.70). These findings are in line with an earlier study which describes the use of surface-based superimposition in individual teeth movement [34].

In this study, the rate of OTM shows a large individual variation. However, from start to end, the individual differences in rate of OTM were maintained. This is the first to report this variability using a sequence of 3D intra-oral scans using an easy-to-use novel method. Large variety in rate of OTM has been reported in other 2D and 3D plaster model studies on OTM as well [6, 21]. Previous studies on orthodontic tooth movement only compared the differences between the pre-treatment and post-treatment position [21, 22]. Obtaining digital models from plaster casts every week is impractical and requires much patient effort, time and is costly [21]. Furthermore, complicated orthodontic appliances may cause distortion and tearing when the impression is removed from the mouth. An intra-oral scanner can directly generate a digital model from the patients’ dentition, but is more expensive and operator-sensitive. Our study shows that the rate of OTM for the first week predicts that of 6 weeks really well, thereby making possible longitudinal studies easier to accomplish.

One of the most notable findings was the positive correlation between S&L and OTM. This indicates that more inflammation leads to a higher rate of OTM or possibly vice versa. This is supported by another study showing that the inflammatory marker myeloperoxidase correlates with the amount of tooth movement [36]. The level of myeloperoxidase was also significantly correlated to the level of cytokine receptor for nuclear factor kappa-B ligand (RANKL). RANKL stimulates osteoclastogenesis and osteoblastogenesis [36]. In this study, the gingival inflammation might also have stimulated myeloperoxidase and RANKL expression, but this was not measured. Also, no significant correlation was found between inflammation and MMPs, while inflammation was expected to increase MMP levels since these are needed for periodontal remodeling [18].

Although not significant, the levels of MMP-2-complex seemed to correlate with OTM. This could indicate a role for this gelatinase in tooth movement. MMP-2 is mainly responsible for the degradation of gelatin, the degradation product of collagen type I, similar to MMP-9. Collagen type I is the main component of the bone ECM. Other MMPs degrading collagen type I (MMPs 1, 8 and 13) could therefore also be investigated in the future. In this study, MMPs were analyzed using GCF of the patients. A common problem in the analysis of GCF is the small volume available [15]. Since the volume of GCF may differ between the sampling moments, the protein concentration was used as a reference. MMPs were analyzed by gelatin zymography [9]. The presence of MMP-2 complexes in GCF could indicate a role for MMP-2 and complexes thereof in OTM in vivo. In another study on GCF from periodontitis patients, both forms of MMP-2 were only detected in GCF from patients but not in healthy subjects [37]. In our gelatin zymograms, a band was also found at 240 kDa, which might represent a multimer of MMP-9 [38]. The lack of a significant correlation between gelatinase levels and OTM raises the question whether gelatinase levels in GCF reflect the actual levels in the PDL and if other MMPs (collagenases) might be more important [37].

Since this study had a limited number of participants with a large variation in malocclusion, it was difficult to calculate a mixed effects model that could be used to predict OTM rate. Other biological markers for bone remodeling in GCF, such as myeloperoxidase and RANKL or IL-1 and TNF-α, might correlate better with OTM [39]. These markers are now used in periodontal but not yet in orthodontic research. These markers are, however, difficult to detect in small samples. To obtain a larger sample of GCF, it is advised to use filter strips instead of a syringe [36].

Inter- or intra-arch obstacles (e.g. neighboring teeth, antagonist) can also play an important role in OTM [40]. In this study, this was not taken into account as the main aim of this study was to prove whether there is a correlation between OTM and the levels of MMPS in GCF. However, as inter- or intra-arch obstacles can play an important role, their influence should be further investigated.

## Conclusion and clinical relevance

In this study, a novel 3D method was used to correlate the rate of OTM to other parameters. Since we hypothesized that the individual OTM rate varies highly, several parameters were investigated. The S&L index was found to have a significant correlation to the rate of OTM. We also hypothesized that using this novel method, a more accurate rate of OTM can be easily determined. A high variation in the rate of OTM was found in this study, but interestingly, individual patients maintained a relatively constant rate of OTM throughout the experimental period. Furthermore, inflammation induces faster orthodontic tooth movement or vice versa. Future studies should include other biological markers related to bone remodeling in order to predict the rate of OTM. Our standardized treatment protocol and efficient 3D method are recommended for future studies. In conclusion, this novel 3D method shows large individual variation in rate of OTM, which could help orthodontic staff and patients to obtain an individualized treatment plan with personalized intervals between the (now standard 6 weeks) appointments.

## References

[1] Schuller AA, van Kampen I, Poorterman J, Verrips G (2013) Kies voor tanden. Een onderzoek naar mondgezondheid en preventief tandheelkundig gedrag van jeugdigen. Hoofdmeting 2011, een vervolg op de reeks TJZ-onderzoeken. Rapportnummer: TNO/LS 2013 R10056

[2] Yao J, Li DD, Yang YQ, McGrath CPJ, Mattheos N (2016). What are patients’ expectations of orthodontic treatment: a systematic review. BMC Oral Health.

[3] Chadwick S (2016). Iatrogenic effects of orthodontic treatment: decision-making in prevention, diagnosis and treatment. J Orthod.

[4] Pilon JJ, Kuijpers-Jagtman AM, Maltha JC (1996). Magnitude of orthodontic forces and rate of bodily tooth movement. An experimental study. Am J Orthod Dentofac Orthop.

[5] Van Leeuwen EJ, Kuijpers-Jagtman AM, Von den Hoff JW, Wagener FADTG, Maltha JC (2010). Rate of orthodontic tooth movement after changing the force magnitude: an experimental study in beagle dogs. Orthod Craniofacial Res.

[6] Jian F et al (2013) Initial arch wires for tooth alignment during orthodontic treatment with fixed appliances. Cochrane Database Syst Rev 2013(4) John Wiley and Sons Ltd 10.1002/14651858.CD007859.pub3 PMC6465075 23633347

[7] Henneman S, Von Den Hoff JW, Maltha JC (2008). Mechanobiology of tooth movement. Eur J Orthod.

[8] Krishnan V, Davidovitch Z (2009). On a path to unfolding the biological mechanisms of orthodontic tooth movement. J Dent Res.

[9] Snoek-van Beurden PAM, Von Den Hoff JW (2005). Zymographic techniques for the analysis of matrix metalloproteinases and their inhibitors. BioTechniques.

[10] Delima AJ, Van Dyke TE (2003). Origin and function of the cellular components in gingival crevice fluid. Periodontol.

[11] Ren Y, Vissink A (2008). Cytokines in crevicular fluid and orthodontic tooth movement. Eur J Oral Sci.

[12] Kumar AA, Saravanan K, Kohila K, Kumar SS (2015). Biomarkers in orthodontic tooth movement. J Pharm Bioallied Sci..

[13] Yamaguchi M, Yoshii M, Kasai K (2006). Relationship between substance P and interleukin-1 β in gingival crevicular fl uid during orthodontic tooth movement in adults. Eur J Orthod.

[14] Dudic A, Kiliaridis S, Mombelli A, Giannopoulou C (2006). Composition changes in gingival crevicular fluid during orthodontic tooth movement: comparisons between tension and compression sides. Eur J Oral Sci.

[15] Bildt MM, Bloemen M, Kuijpers-Jagtman AM, Von Den Hoff JW (2009). Matrix metalloproteinases and tissue inhibitors of metalloproteinases in gingival crevicular fluid during orthodontic tooth movement. Eur J Orthod.

[16] Vandooren J, Geurts N, Martens E, Van Den Steen PE, Opdenakker G (2013). Zymography methods for visualizing hydrolytic enzymes. Nat Methods.

[17] Takahashi I, Nishimura M, Onodera K, Bae JW, Mitani H, Okazaki M, Sasano Y, Mitani H (2003). Expression of MMP-8 and MMP-13 genes in the periodontal ligament during tooth movement in rats. J Dent Res.

[18] Ingman T, Apajalahti S, Mäntylä P, Savolainen P, Sorsa T (2005). Matrix metalloproteinase-1 and -8 in gingival crevicular fluid during orthodontic tooth movement: a pilot study during 1 month of follow-up after fixed appliance activation. Eur J Orthod.

[19] Cantarella G, Cantarella R, Caltabiano M, Risuglia N, Bernardini R, Leonardi R (2006). Levels of matrix metalloproteinases 1 and 2 in human gingival crevicular fluid during initial tooth movement. Am J Orthod Dentofac Orthop.

[20] Martin CB, Chalmers EV, McIntyre GT, Cochrane H, Mossey PA (2015). Orthodontic scanners: what’s available?. J Orthod.

[21] Yun D, Choi DS, Jang I, Cha BK (2018). Clinical application of an intraoral scanner for serial evaluation of orthodontic tooth movement: a preliminary study. Korean J Orthod.

[22] Giannopoulou C, Dudic A, Pandis N, Kiliaridis S (2016). Slow and fast orthodontic tooth movement: an experimental study on humans. Eur J Orthod.

[23] Power calculator for continuous outcome superiority trial, Sealed Envelope Ltd., 2012. [Online]. Available: www.sealedenvelope.com/power/continuous-superiority.

[24] Francisconi MF, Janson G, Henriques JFC, de Freitas KMS (2016). Evaluation of the force generated by gradual deflection of orthodontic wires in conventional metallic, esthetic, and self-ligating brackets. J Appl Oral Sci.

[25] Silness J, Löe H (1964). Periodontal disease in pregnancy II. Correlation between oral hygiene and periodontal condition. Acta Odontol Scand.

[26] Handem RH et al (2016) External root resorption with the self-ligating damon system—a retrospective study. Prog Orthod 17(1) 10.1186/s40510-016-0133-1 PMC4929110 27365168

[27] Cuperus AMR, Harms MC, Rangel FA, Bronkhorst EM, Schols JGJH, Breuning KH (2012). Dental models made with an intraoral scanner: a validation study. Am J Orthod Dentofac Orthop.

[28] Nedelcu R, Olsson P, Nyström I, Rydén J, Thor A (2018). Accuracy and precision of 3 intraoral scanners and accuracy of conventional impressions: a novel in vivo analysis method. J Dent.

[29] Deferm JT, Schreurs R, Baan F, Bruggink R, Merkx MAW, Xi T, Bergé SJ, Maal TJJ (2018). Validation of 3D documentation of palatal soft tissue shape, color, and irregularity with intraoral scanning. Clin Oral Investig.

[30] Nada RM, Maal TJJ, Breuning KH, Bergé SJ, Mostafa YA, Kuijpers-Jagtman AM (2011) Accuracy and reproducibility of voxel based superimposition of cone beam computed tomography models on the anterior cranial base and the zygomatic arches. PLoS One 6(2) 10.1371/journal.pone.0016520 PMC3036654 21347419

[31] Ongkosuwito EM, Goos JAC, Wattel E, Van Der Wal KGH, Van Adrichem LNA, Van Neck JW (2012). Assessment of volumetric changes with a best-fit method in three-dimensional stereophotograms. Cleft Palate-Craniofacial J.

[32] Choi DS, Jeong YM, Jang I, Jost-Brinkmann PG, Cha BK (2010). Accuracy and reliability of palatal superimposition of three-dimensional digital models. Angle Orthod.

[33] Baan F et al (2016) A new 3D tool for assessing the accuracy of bimaxillary surgery: the OrthoGnathicanAlyser. PLoS One 11(2) 10.1371/journal.pone.0149625 PMC4762705 26901524

[34] Baan F, de Waard O, Bruggink R, Xi T, Ongkosuwito EM, Maal TJJ (2019). Virtual setup in orthodontics: planning and evaluation.

[35] Bates D, Mächler M, Bolker B, Walker S (2015). Fitting linear mixed-effects models using lme4 | Bates | Journal of Statistical Software. J Stat Softw.

[36] Saloom HF, Papageorgiou SN, Carpenter GH, Cobourne MT (2017). Impact of obesity on orthodontic tooth movement in adolescents: a prospective clinical cohort study. J Dent Res.

[37] Bildt MM, Bloemen M, Kuijpers-Jagtman AM, Von den Hoff JW (2008). Collagenolytic fragments and active gelatinase complexes in periodontitis. J Periodontol.

[38] Goldberg GI, Strongin A, Collier IE, Genrich LT, Marmer BL (1992). Interaction of 92-kDa type IV collagenase with the tissue inhibitor of metalloproteinases prevents dimerization, complex formation with interstitial collagenase, and activation of the proenzyme with stromelysin. J Biol Chem.

[39] Baeza M, Garrido M, Hernández-Ríos P, Dezerega A, García-Sesnich J, Strauss F, Aitken JP, Lesaffre E, Vanbelle S, Gamonal J, Brignardello-Petersen R, Tervahartiala T, Sorsa T, Hernández M (2016). Diagnostic accuracy for apical and chronic periodontitis biomarkers in gingival crevicular fluid: an exploratory study. J Clin Periodontol.

[40] Dudic A, Giannopoulou C, Kiliaridis S (2013). Factors related to the rate of orthodontically induced tooth movement. Am J Orthod Dentofac Orthop.

